# On Being a “Physician Patient” with His Own Experimental Therapeutic Drug

**DOI:** 10.4274/tjh.2018.0254

**Published:** 2018-11-13

**Authors:** Rafiye Çiftçiler, İbrahim C. Haznedaroğlu

**Affiliations:** 1 Hacettepe University Faculty of Medicine, Department of Hematology, Ankara, Turkey

**Keywords:** Ankaferd, Burn, Physician patient, Mucosal healing

## To the Editor,

We have read with great interest the paper by Patıroğlu et al. [1] on the mucosal healing effects of Ankaferd BloodStopper (ABS), recently published in this journal. They suggested that ABS could be effective for the management of chemotherapy-induced mucositis. We would like to share our own experience with ABS on the burn-induced skin wounds of a patient.

The patient herein is a physician and the senior author of this paper (İ.C.H.). He is also the mentor of the first author (R.Ç.). The experimental therapeutic drug is ABS, which was developed as a medicine with numerous clinical studies (https://www.ncbi.nlm.nih.gov/m/pubmed/?term=ankaferd), mostly authored by İ.C.H. himself. ABS is the first topical hemostatic agent acting on red blood cells and fibrinogen gamma interactions to be tested in clinical trials [2]. ABS is a drug officially approved for the management of clinical hemorrhages in Turkey [3]. However, ABS has never been used in humans for the therapy of burns until İ.C.H. had his left forearm severely burned by a boiling tea kettle. In his physical examination, the burnt areas had acutely developed heavy erythematous lesions, which then complicated into several bullous lesions.

At the time of the burn accident, ABS for burn wound management had only been demonstrated in rats [4,5]. The burns were induced in Wistar albino rats by Kaya et al. [4] they showed that ABS decreases the inflammation and wound diameters and increases the wound contraction and tissue fibrosis in rats with burn injuries. The results of another rat study demonstrated that ABS has a positive effect on second-degree thermal burn healing [6].

**Figure 1:**
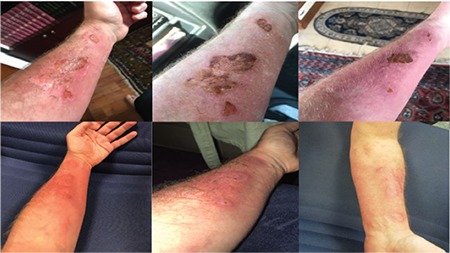
The senior author’s burn wounds before and after treatment with Ankaferd BloodStopper.

The emergency state of the severe burn lesions and the availability of ABS at the time of the accident enabled us to apply it topically to the burn lesions of İ.C.H. The burn lesions were clearly regressed and wound healing occurred with no complications upon the usage of ABS in our physician patient (Figure 1).

In the history of medical science, there are many inventors that applied their own therapeutic tools for the management of their own diseases, such as Dr. Barry J. Marshall. He drank *Helicobacter pylori* bacteria himself and developed stomach ulcers within a few days. He later successfully treated himself with antibiotics and went on to win the Nobel Prize [7]. 

The conclusions that were drawn from our unique clinical story are as follows:

- Medical inventors and researchers are enthusiastic for the use of their experimental drugs in clinical situations.

- The weakest aspect of evidence-based medicine is the ‘lack of evidence’ in the related particular clinical problem. This represents a great challenge, particularly for real-life medical emergencies.

- Sometimes medical doctors have to make clinical decisions despite the lack of solid scientific evidence in the presence of urgent medical needs.

- Rat and animal studies may be the only source of evidence for human use in some medical emergencies.

- Nevertheless, the best clinical practice should rely on the best current evidence obtained through randomized controlled clinical trials.
